# From static labels to dynamic trajectories: MASLD–MetALD–ALD as a dynamic continuum within the framework of steatotic liver disease

**DOI:** 10.1136/egastro-2026-100430

**Published:** 2026-05-22

**Authors:** Lanlan Chen, Paul Horn, Frank Tacke

**Affiliations:** 1 Department of Hepatology and Gastroenterology, Campus Virchow-Klinikum (CVK) and Campus Charité Mitte (CCM), Charité - Universitätsmedizin Berlin, Berlin, Germany; 2 Department of Hepatobiliary and Pancreatic Surgery, General Surgery Center, the First Hospital of Jilin University, Changchun, People's Republic of China; 3 Berlin Institute of Health at Charité – Universitätsmedizin Berlin, BIH Biomedical Innovation Academy, BIH Charité Digital Clinician Scientist Program, Berlin, Germany

**Keywords:** Fatty Liver, Non-alcoholic Fatty Liver Disease, Liver Diseases, Alcoholic, Alcohol-Related Disorders, Alcohol Drinking

## Introduction

 Steatotic liver disease (SLD) is an umbrella term mainly encompassing metabolic dysfunction-associated steatotic liver disease (MASLD), metabolic dysfunction and alcohol-associated liver disease (MetALD) and alcohol-associated liver disease (ALD).^1^ This redefinition goes beyond terminology and reflects biological reality as these entities represent a continuous spectrum rather than fixed diagnoses, arising from two dynamic exposures (ie, alcohol intake and metabolic dysfunction) that fluctuate over time and enable interconversion.^2^

**Figure 1:**
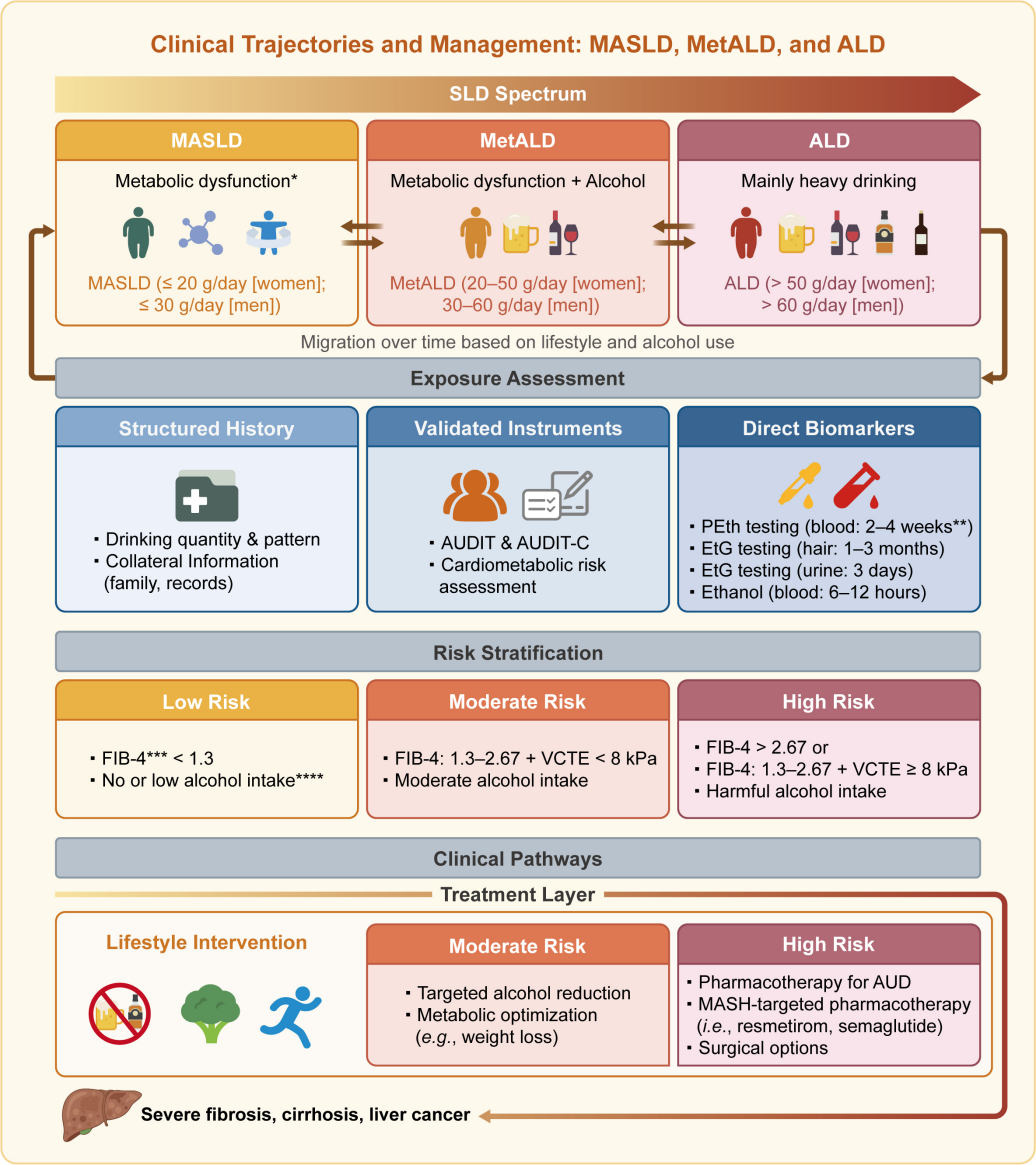
Clinical trajectories, diagnostic framework and risk-stratified management across the MASLD–MetALD–ALD spectrum. This schematic depicts the continuum of SLD, including MASLD, MetALD and ALD, with dynamic transitions driven by metabolic factors and alcohol use. Diagnosis integrates structured history, validated tools (AUDIT/AUDIT-C) and direct biomarkers such as PEth in blood and EtG in hair to estimate recent alcohol intake. Risk stratification relies on non-invasive fibrosis assessment: low risk (FIB-4 <1.3), moderate risk (FIB-4 of 1.3–2.67 plus VCTE <8 kPa) and high risk (FIB-4 >2.67 or FIB-4 of 1.3–2.67 plus VCTE ≥8 kPa). Management is risk-based, combining alcohol reduction or abstinence and metabolic optimisation. Advanced disease may require pharmacotherapy, bariatric surgery or liver transplantation, whereas disease progression may potentially lead to cirrhosis and hepatocellular carcinoma. *Metabolic dysfunction includes obesity, type 2 diabetes or hyperglycaemia, dyslipidaemia (high triglycerides and/or low high-density lipoprotein cholesterol) and arterial hypertension. **The suggested cut-off values of PEth are: MASLD <20 ng/mL; MetALD: 20–200 ng/mL; ALD >200 ng/mL according to expert review. ***The threshold for FIB-4 is >2.0 in individuals aged >65 years. ****Alcohol intake should be assessed as a cumulative value not only based on the recent self-reports. ALD, alcohol-associated liver disease; AUD, alcohol use disorder; AUDIT, alcohol use disorders identification test; AUDIT-C, alcohol use disorders identification test-consumption; EtG, ethyl glucuronide; FIB-4, fibrosis-4; MASH, metabolic dysfunction-associated steatohepatitis; MASLD, metabolic dysfunction-associated steatotic liver disease; MetALD, metabolic dysfunction and alcohol-associated liver disease; PEth, phosphatidylethanol; SLD, steatotic liver disease; VCTE, vibration-controlled transient elastography.

MetALD is currently positioned between MASLD and ALD and is defined by alcohol dose thresholds (figure 1). It is conceptually important because it reflects real-world clinical overlap.^3^ A recent study emphasised that MetALD, together with ALD, is independently associated with higher risks of all-cause mortality and liver-related event (LRE) compared with the non-SLD group.^4^ However, clinical outcomes (ie, all-cause mortality and LRE) did not differ significantly between the MASLD and non-SLD groups.^4^ One possible explanation is that simple hepatic steatosis may be seen as a mere phenotype, not necessarily constituting a disease entity like metabolic dysfunction-associated steatohepatitis (MASH) or liver fibrosis and may not be closely associated with prognosis.^5^

Thus, SLD subclassification discretises an inherently dynamic process as alcohol use and metabolic dysfunction fluctuate over time. Current classification reflects only a time-point assessment, requiring longitudinal reassessment rather than static labelling (figure 1).

## Why interconversion is expected: two dynamic indicators, one shared liver phenotype

Lifetime alcohol history is highly variable, and binge drinking patterns add complexity. Clinically, alcohol exposure may shift across diagnostic thresholds over time, leading to transitions among MASLD, MetALD and ALD.^3^ If subclass labels are used without a plan for reassessment, clinicians may mistake a transient state for a stable disease entity. Metabolic risk factors are similarly dynamic and modifiable. This means that a patient’s ‘metabolic load’ is not fixed, and neither is the biological consequence of a given amount of alcohol. Mechanistically, both exposures converge on shared pathogenic pathways (ie, lipotoxicity, oxidative stress) with reciprocal interactions amplifying disease progression.^3^ Alcohol consumption can directly exacerbate metabolic risk factors by promoting weight gain, hypertriglyceridaemia and elevated blood pressure, thereby amplifying metabolic dysfunction; conversely, underlying metabolic dysfunction may increase susceptibility to alcohol-induced liver injury. These reciprocal interactions provide a biological basis for the synergistic and dynamic nature of disease progression observed in MetALD.^3^

A prospective cohort demonstrated substantial migration across SLD subclasses over time, confirming the dynamic nature of disease classification.^6^ At the 6-month follow-up, 36% of individuals originally classified as MetALD had shifted subclass, primarily to MASLD (19%) or ALD (17%). Similarly, 32% of those initially classified as ALD transitioned to MetALD (21%) or MASLD (11%).^6^ In contrast, MASLD was more stable, with only 11% of individuals reclassified at 6 months.^6^ These findings highlight the fluidity of SLD subclasses, driven by changes in alcohol intake and metabolic risk, and underscore the need for longitudinal reassessment, particularly in clinical trial settings and treatment planning (figure 1).

The fluidity of SLD disease categories with changing alcohol drinking patterns uncovers a fundamental issue with the current classification system: The diagnoses of MASLD, MetALD and ALD rely heavily on the assessment of current alcohol intake. However, most clinicians will likely consider cumulative lifetime exposure to determine the impact of alcohol consumption on the development of a patient’s liver disease: A patient with diabetes and SLD who has drunk heavily for more than 20 years would not suddenly be reclassified as MASLD when they stop drinking. Unfortunately, while relying more on the history of alcohol consumption would probably stabilise disease categories over time, adequate universal thresholds for cumulative alcohol intake do not exist and alcohol-related risks are strongly modified by sex, age and metabolic status.

## The bottleneck: alcohol exposure is frequently mismeasured

A dynamic framework collapses if its defining exposure is poorly measured. In clinical settings, accurate evaluation of alcohol intake is challenging: self-reported alcohol intake may be under-reported by up to 57.7%, and only one-third of self-reported cases align with objective measures.^3^ Under-ascertainment of alcohol use may distort risk stratification, delay identification of harmful drinking or alcohol use disorder (AUD) and bias clinical research. Additionally, the variability of alcohol intake assessment can lead to inconsistent estimates of alcohol exposure and limit comparability across cohorts. Standardisation of alcohol assessment methods, including the adoption of validated core instruments such as Timeline Followback, is therefore essential to improve consistency across studies and to enable reliable estimation of disease burden and outcomes.^7^ Thus, in the context of SLD, objective alcohol assessment is increasingly recognised as a critical component of clinical practice.

## Objective assessment of alcohol intake as a key component of SLD management

Phosphatidylethanol (PEth), a blood-based biomarker less affected by sex or body mass index, reflects the quantity of alcohol consumption over the preceding 1–3 weeks.^8^ Current expert consensus indicates that concentrations<20 ng/mL effectively exclude clinically relevant alcohol intake, levels between 20–200 ng/mL are indicative of moderate consumption (consistent with MetALD) and values ≥200 ng/mL suggest harmful drinking.^9^ In addition to PEth, ethyl glucuronide in hair can help to assess harmful alcohol intake for even up to 3 months and in urine for 3 days.^8^ Compared with questionnaire-based screening tools, these biomarkers demonstrate superior sensitivity for detecting recent alcohol use and are particularly valuable in identifying individuals whose reported intake falls within the diagnostic ‘gray zone’ between MASLD and MetALD.

However, interpretation of PEth results requires caution, as false-positive findings may arise in the context of confounding factors such as recent blood transfusion or the use of ethanol-containing medications, which can introduce PEth precursors into circulation.^8^ Moreover, more evidence is needed to accurately calibrate the threshold of PEth when discriminating MASLD, MetALD and ALD across ethnicities and different age groups. Besides, the limitations of PEth should be acknowledged and addressed in the future, including variability in access, lack of assay standardisation and evolving cut-off values across populations. Collectively, integrating validated screening questionnaires with direct biomarkers, particularly PEth, provides a robust, evidence-based framework for accurately quantifying alcohol exposure in both clinical practice and SLD-related research settings. Importantly, PEth should complement, rather than replace, careful history taking and clinical judgement^3^ (figure 1).

## A reframed clinical priority: identifying harmful drinking and AUD before defining MetALD

Simple hepatic steatosis alone is not associated with overall or liver-specific mortality, but is clearly linked to cardiometabolic risk factors, whereas liver fibrosis is a key determinant of mortality in patients with SLD, and fibrosis regression is usually a primary endpoint in clinical trials serving as a surrogate for expected long-term benefit.^5^ Furthermore, liver fibrosis and related major adverse liver outcomes (ie, cirrhosis, hepatocellular carcinoma, liver transplantation, and liver-related death) are more frequent in patients with MetALD and ALD than in patients with MASLD. Therefore, it may be more important to screen and identify harmful drinking and diagnose AUD (when appropriate) in patients with SLD first, and then define MetALD accordingly, rather than the other way around.

If SLD nomenclature is intended to improve outcomes, it should operationally prioritise patients to the right care pathways, not merely rename categories (figure 1). Current SLD subclassification is based primarily on recent alcohol exposure, whereas cumulative lifetime exposure, although clinically relevant, is not yet standardised for formal classification. Distinguishing these two dimensions is essential to avoid conceptual ambiguity and to align clinical reasoning with existing nomenclature. Accordingly, we propose that at each follow-up the patient’s record could explicitly include: (1) SLD subclass (MASLD, MetALD or ALD) based on past and current alcohol exposure and metabolic criteria; (2) fibrosis stage (no or mild fibrosis vs moderate, advanced fibrosis or cirrhosis), as an effect modifier of risk and management intensity; (3) trajectory markers, including current alcohol intake (ie, weekly grams, binge episodes, PEth or other alcohol biomarkers when indicated and AUD status) and metabolic (ie, weight trend, glycaemic control, lipid or blood pressure control and therapies) characteristics. This approach converts the SLD framework from a naming system into a longitudinal risk-management system. A key unmet need is the development of standardised, longitudinal criteria to define transitions among MASLD, MetALD and ALD. Future studies should integrate repeated measures of alcohol exposure, metabolic status and liver-related outcomes to establish evidence-based thresholds that capture clinically meaningful trajectory shifts. Such efforts will be essential to translate the dynamic SLD framework into both clinical practice and trial design.

## Conclusion

With the evolving SLD framework, MASLD, MetALD and ALD should not be viewed as static labels. A trajectory-first approach, integrating longitudinal reassessment and objective alcohol measurement, may better reflect disease biology and guide clinical decision-making.

## Data Availability

Data sharing not applicable as no datasets generated and/or analysed for this study.
