# Supplementary material for: From static labels to dynamic trajectories: MASLD–MetALD–ALD as a dynamic continuum within the framework of steatotic liver disease
Source: eGastroenterology. 2026 May 22;4(2):e100430. doi: 10.1136/egastro-2026-100430 (PMC13202102; doi:10.1136/egastro-2026-100430)
Supplement: Supplementary file 1 [file egastro-2026-100430.reviewer_comments.pdf]

PEER REVIEW HISTORY

eGastroenterology publishes all reviews undertaken for accepted manuscripts. Reviewers are asked to complete a checklist review form and are provided with free text boxes to elaborate on their assessment. These free text comments are reproduced below.

ARTICLE DETAILS

**TITLE (PROVISIONAL)**	From static labels to dynamic trajectories: MASLD – MetALD - ALD as a dynamic continuum within the framework of steatotic liver disease
**AUTHORS**	Chen, Lanlan; Horn, Paul; Tacke, Frank

VERSION 1 - REVIEW

**REVIEWER NAME**	***Arab, Juan Pablo***
**REVIEWER AFFILIATION**	Virginia Commonwealth University School of Medicine, Division of Gastroenterology, Hepatology, and Nutrition
**REVIEWER CONFLICT OF INTEREST**	No
**AI DISCLOSURE**	No, I have not used AI
**DATE REVIEW RETURNED**	14-Apr-2026

**GENERAL COMMENTS**	This editorial provides a timely and clinically relevant perspective on steatotic liver disease by arguing that MASLD, MetALD, and ALD should be viewed as dynamic states rather than fixed labels. The manuscript's main strengths are its clear conceptual framing, its emphasis on longitudinal reassessment, and its practical focus on improving alcohol exposure assessment through direct biomarkers such as PEth. The proposed "trajectory-first" approach is thoughtful, clinically meaningful, and likely to be useful for both routine care and future trial design.<br><br>Comments for the authors<br>1. Clarify and harmonize alcohol threshold definitions throughout the text and figure.<br>The manuscript is generally clear, but the alcohol thresholds should be checked carefully for internal consistency. In the Introduction, the daily and weekly thresholds are presented, but one sentence appears to state ALD as ">60 g/week (men)," which seems inconsistent with the surrounding framework and likely needs correction. The terminology in the figure should also be harmonized, including "man/men," inequality symbols, and formatting of the MetALD range. This is a minor editorial issue, but important because the paper's central argument depends on precise subclass definitions.<br>2. Temper some of the more prescriptive language around PEth and "standard of care."<br>The section on objective alcohol assessment is strong, but statements such as objective assessment being "not optional" or implying PEth-based adjudication as standard of care may read as somewhat stronger than the currently available evidence and real-world implementation allow. It would improve balance to acknowledge more explicitly that PEth is highly valuable, especially

	in high-stakes or uncertain cases, but that access, assay standardization, and optimal cutoffs across settings remain evolving.<br>3. Better distinguish current alcohol exposure from lifetime/cumulative exposure in the proposed framework.<br>One of the best points in the editorial is that current SLD subclassification is highly dynamic, whereas clinicians often also rely on cumulative lifetime alcohol burden. However, the manuscript could sharpen this distinction further by explicitly stating that current nomenclature is based largely on recent exposure, while lifetime exposure remains clinically important but insufficiently standardized for formal classification. This would strengthen the manuscript's key conceptual message and avoid confusion between what is recommended pragmatically versus what is currently codified.<br>4. Refine the figure/table so the practical algorithm is even easier to apply.<br>Figure 1 and the summary box are useful, but a few elements could be simplified for usability. For example, the fibrosis risk strata and the linkage between FIB-4, VCTE, alcohol biomarkers, and resulting care pathways could be made more linear and visually cleaner. In addition, some labels in the figure are crowded or phrased inconsistently. A modest visual revision would enhance readability and make the proposed framework more immediately translatable to clinical practice.

**REVIEWER NAME**	***Zheng, Ming-Hua***
**REVIEWER AFFILIATION**	The First Affiliated Hospital of Wenzhou Medical University, MAFLD Research Center, Department of Hepatology
**REVIEWER CONFLICT OF INTEREST**	No
**AI DISCLOSURE**	No, I have not used AI
**DATE REVIEW RETURNED**	15-Apr-2026

**GENERAL COMMENTS**	This editorial redefines steatotic liver disease as a dynamic continuum rather than static categories. It highlights frequent transitions between MASLD, MetALD, and ALD driven by changes in alcohol consumption and metabolic status. The authors appropriately emphasize the limitations of self-reported alcohol intake and advocate for the use of objective biomarkers such as PEth. They also propose a clinically practical framework—prioritizing screening for harmful drinking and alcohol use disorder (AUD), followed by subtype classification and longitudinal assessment alongside fibrosis staging. Overall, this work provides an important conceptual shift toward personalized, trajectory-based management that may improve both clinical care and trial design in steatotic liver disease.<br><br>I only have a few minor suggestions:<br><br>1. The manuscript could be further strengthened by addressing the need for standardized, longitudinal criteria to classify transitions between MASLD, MetALD, and ALD. While the dynamic nature of SLD is well articulated, most supporting evidence remains cross-sectional, and large-scale longitudinal data capturing changes in alcohol exposure, metabolic status, and liver outcomes are still limited. A brief discussion on how future studies could define evidence-based thresholds would enhance the translational relevance of the proposed trajectory model.

	2. The authors may further elaborate on approaches to improve the accuracy and consistency of alcohol exposure assessment. Although biomarkers such as PEth represent an important advance, their cut-offs may vary across populations, age groups, and ethnicities. Discussion of standardized strategies, or combined approaches integrating biomarkers with clinical assessment, would strengthen the methodological framework for dynamic classification and risk stratification. The authors may also consider referencing recent work in this area (e.g., PMID: 41831477).<br><br>3. The manuscript would also benefit from a more detailed discussion of the biological interplay between metabolic dysfunction and alcohol exposure. Clarifying how these factors jointly drive lipotoxicity, inflammation, and fibrosis could help inform mechanism-based therapies and improve personalized risk prediction.

VERSION 1 – AUTHOR RESPONSE

Reviewer: 1

Comments to the Author

This editorial provides a timely and clinically relevant perspective on steatotic liver disease by arguing that MASLD, MetALD, and ALD should be viewed as dynamic states rather than fixed labels. The manuscript's main strengths are its clear conceptual framing, its emphasis on longitudinal reassessment, and its practical focus on improving alcohol exposure assessment through direct biomarkers such as PEth. The proposed "trajectory-first" approach is thoughtful, clinically meaningful, and likely to be useful for both routine care and future trial design.

Comments for the authors

1. Clarify and harmonize alcohol threshold definitions throughout the text and figure.

The manuscript is generally clear, but the alcohol thresholds should be checked carefully for internal consistency. In the Introduction, the daily and weekly thresholds are presented, but one sentence appears to state ALD as ">60 g/week (men)," which seems inconsistent with the surrounding framework and likely needs correction. The terminology in the figure should also be harmonized, including "man/men," inequality symbols, and formatting of the MetALD range. This is a minor editorial issue, but important because the paper's central argument depends on precise subclass definitions.

Reply 1: Thank you for identifying this important inconsistency. We have carefully reviewed and harmonized all alcohol threshold definitions throughout the manuscript and figure. We decided to delete the description of alcohol intake range as it was clearly displayed in the Figure 1. Also, we have revised the figure to ensure consistent terminology (e.g., "men" instead of "man"), inequality symbols, and formatting.

2. Temper some of the more prescriptive language around PEth and "standard of care."

The section on objective alcohol assessment is strong, but statements such as objective assessment being "not optional" or implying PEth-based adjudication as standard of care may read as somewhat

stronger than the currently available evidence and real-world implementation allow. It would improve balance to acknowledge more explicitly that PEth is highly valuable, especially in high-stakes or uncertain cases, but that access, assay standardization, and optimal cutoffs across settings remain evolving.

Reply 2: We appreciate this important suggestion and have revised the text to adopt a more balanced tone. Statements such as “not optional” have been replaced with more measured wording (e.g., “increasingly recognized as a critical component”). We now emphasize that objective alcohol assessment, including PEth, is particularly valuable in high-stakes or uncertain clinical scenarios. We have explicitly acknowledged current limitations, including variability in access, lack of assay standardization, and evolving cut-off values across populations.

3. Better distinguish current alcohol exposure from lifetime/cumulative exposure in the proposed framework.

One of the best points in the editorial is that current SLD subclassification is highly dynamic, whereas clinicians often also rely on cumulative lifetime alcohol burden. However, the manuscript could sharpen this distinction further by explicitly stating that current nomenclature is based largely on recent exposure, while lifetime exposure remains clinically important but insufficiently standardized for formal classification. This would strengthen the manuscript’s key conceptual message and avoid confusion between what is recommended pragmatically versus what is currently codified.

Reply 3: We agree and have strengthened this conceptual distinction in the revised manuscript. We have clarified that: current SLD subclassification is based primarily on recent alcohol exposure, whereas cumulative lifetime exposure, although clinically relevant, is not yet standardized for formal classification. We further emphasize that distinguishing these two dimensions is essential to avoid conceptual ambiguity and to align clinical reasoning with existing nomenclature.

4. Refine the figure/table so the practical algorithm is even easier to apply.

Figure 1 and the summary box are useful, but a few elements could be simplified for usability. For example, the fibrosis risk strata and the linkage between FIB-4, VCTE, alcohol biomarkers, and resulting care pathways could be made more linear and visually cleaner. In addition, some labels in the figure are crowded or phrased inconsistently. A modest visual revision would enhance readability and make the proposed framework more immediately translatable to clinical practice.

Reply 4: Thank you for this helpful suggestion and have revised the figure accordingly.

The figure has been restructured into: Exposure assessment (alcohol + metabolic factors); Risk stratification (FIB-4, VCTE); Clinical pathways (risk-adapted management). We have simplified labels, reduced visual crowding, and harmonized terminology to improve readability and practical applicability. The table 1 was deleted to reduce redundancy.

Reviewer: 2

Comments to the Author

This editorial redefines steatotic liver disease as a dynamic continuum rather than static categories. It highlights frequent transitions between MASLD, MetALD, and ALD driven by changes in alcohol consumption and metabolic status. The authors appropriately emphasize the limitations of self-reported alcohol intake and advocate for the use of objective biomarkers such as PEth. They also propose a clinically practical framework—prioritizing screening for harmful drinking and alcohol use disorder (AUD), followed by subtype classification and longitudinal assessment alongside fibrosis

staging. Overall, this work provides an important conceptual shift toward personalized, trajectory-based management that may improve both clinical care and trial design in steatotic liver disease.

I only have a few minor suggestions:

1. The manuscript could be further strengthened by addressing the need for standardized, longitudinal criteria to classify transitions between MASLD, MetALD, and ALD. While the dynamic nature of SLD is well articulated, most supporting evidence remains cross-sectional, and large-scale longitudinal data capturing changes in alcohol exposure, metabolic status, and liver outcomes are still limited. A brief discussion on how future studies could define evidence-based thresholds would enhance the translational relevance of the proposed trajectory model.

Reply 1: Thank you for this insightful comment. We agree that the development of standardized longitudinal criteria to define transitions across MASLD, MetALD, and ALD is an important unmet need. In the revised manuscript, we have expanded the discussion to explicitly highlight the limitations of current cross-sectional classification and the necessity of longitudinal data integrating repeated alcohol exposure, metabolic status, and liver-related outcomes. We further outline future research directions aimed at establishing evidence-based thresholds for clinically meaningful trajectory shifts.

2. The authors may further elaborate on approaches to improve the accuracy and consistency of alcohol exposure assessment. Although biomarkers such as PEth represent an important advance, their cut-offs may vary across populations, age groups, and ethnicities. Discussion of standardized strategies, or combined approaches integrating biomarkers with clinical assessment, would strengthen the methodological framework for dynamic classification and risk stratification. The authors may also consider referencing recent work in this area (e.g., PMID: 41831477).

Reply 2: We appreciate this important suggestion, highlighting recent advances in this area. We have expanded the section on alcohol exposure assessment to emphasize that a combined approach integrating self-reported intake, validated screening tools, and direct biomarkers provides the most robust framework. In addition, we now explicitly acknowledge the substantial heterogeneity and methodological limitations in alcohol assessment, as highlighted in recent literature. For example, recent work has demonstrated considerable discordance between self-reported alcohol intake and objective biomarkers such as phosphatidylethanol (PEth), raising concerns regarding misclassification across SLD subtypes and the validity of current definitions. We further note that variability in questionnaire design, standard drink definitions, and data collection methods across cohorts can substantially affect alcohol exposure estimates and limit comparability across studies. Moreover, we highlight that biomarker thresholds may vary across populations, underscoring the need for standardization and population-specific validation.

3. The manuscript would also benefit from a more detailed discussion of the biological interplay between metabolic dysfunction and alcohol exposure. Clarifying how these factors jointly drive lipotoxicity, inflammation, and fibrosis could help inform mechanism-based therapies and improve personalized risk prediction.

Reply 3: This is a valuable suggestion. We agree that, beyond shared pathogenic pathways, alcohol exposure and metabolic dysfunction can also interact bidirectionally. In the revised manuscript, we have expanded this section to emphasize that alcohol not only contributes to liver injury through

overlapping mechanisms such as oxidative stress and inflammation, but also directly exacerbates metabolic risk factors, including weight gain, hypertriglyceridemia, and hypertension. Conversely, metabolic dysfunction may increase susceptibility to alcohol-related liver injury. These reciprocal interactions further support the concept of a dynamic and synergistic disease continuum in SLD.

VERSION 2 - REVIEW

**REVIEWER NAME**	***Arab, Juan Pablo***
**REVIEWER AFFILIATION**	Virginia Commonwealth University School of Medicine, Division of Gastroenterology, Hepatology, and Nutrition
**REVIEWER CONFLICT OF INTEREST**	No
**AI DISCLOSURE**	No, I have not used AI
**DATE REVIEW RETURNED**	23-Apr-2026

**GENERAL COMMENTS**	Thank you for addressing my comments. No further comments.

**REVIEWER NAME**	***Zheng, Ming-Hua***
**REVIEWER AFFILIATION**	The First Affiliated Hospital of Wenzhou Medical University, MAFLD Research Center, Department of Hepatology
**REVIEWER CONFLICT OF INTEREST**	No
**AI DISCLOSURE**	No, I have not used AI
**DATE REVIEW RETURNED**	26-Apr-2026

**GENERAL COMMENTS**	This revised version is perfect, no more comments for it now.
